# Seroprevalence of *Toxocara canis* infection and associated risk factors among primary schoolchildren in rural Southern Thailand

**DOI:** 10.1186/s41182-020-00211-0

**Published:** 2020-04-22

**Authors:** Nonthapan Phasuk, Chuchard Punsawad

**Affiliations:** School of Medicine, Walailak University, Nakhon Si Thammarat, Thailand

**Keywords:** Seroprevalence, *Toxocara canis*, Schoolchildren, southern Thailand

## Abstract

**Background:**

Human toxocariasis is a parasitic zoonosis caused by a parasite in the genus *Toxocara* and is transmitted mainly by the accidental ingestion of embryonated *Toxocara canis* (dog roundworm) or *T. cati* (cat roundworm) eggs. Several studies reported that children were the main population at risk for *T. canis* infection. Currently, no reports on the seroprevalence of *T. canis* infection in Thailand are available, and its status remains unknown among children who live in rural areas of southern Thailand.

**Objective:**

This study aimed to investigate the seroprevalence of *T. canis* infection and its associated risk factors among primary schoolchildren in rural areas of Nakhon Si Thammarat Province, Thailand.

**Methods:**

A total of 177 schoolchildren between 6 and 13 years of age were recruited between June and July 2019. Serum anti-*T. canis* IgG antibodies were detected with a commercial ELISA kit. A questionnaire administered by direct interviews was used to collect demographic and behavioral risk factor data.

**Results:**

The overall seroprevalence of *T. canis* infection was 58.2% (103 of 177). The univariate analysis revealed that schoolchildren who did not practice handwashing before a meal (crude odds ratio (COR) = 3.67, 95% CI 1.93–6.95, *P* < 0.001), did not practice hand washing after animal contact (COR = 2.89, 95% CI 1.53–5.47, *P* = 0.001), and drank untreated water (COR = 1.87, 95% CI 1.00–3.48, *P* = 0.049) had an increased risk of acquiring *T. canis* infection. However, after adjusting for confounders, only a lack of handwashing before a meal remained a significant risk factor (adjusted odds ratio (AOR) = 2.20, 95% CI 1.11–4.34, *P* = 0.023). Age, sex, owning a dog, and eating fresh vegetables were not significantly associated with *T. canis* infection in the current study.

**Conclusions:**

This is the first serological investigation of *T. canis* infection among schoolchildren in Thailand. The high rate of *Toxocara* seropositivity reflected high levels of exposure to *T. canis* among schoolchildren in rural areas of southern Thailand. The results also provide baseline data regarding modifiable risk behaviors for effective *T. canis* infection prevention strategies in southern Thailand, especially strengthening hand washing practices among schoolchildren.

## Introduction

Human toxocariasis is one of most prevalent parasitic zoonoses worldwide; it is particularly present in subtropical and tropical regions and in developing countries [1]. Toxocariasis is caused by the larvae of *Toxocara canis* or *Toxocara cati*, whose definitive hosts are dogs and cats, respectively [2]. The global prevalence of *Toxocara* infection in dogs is 11.1% [3]. Humans acquire *Toxocara* spp. via the accidental consumption of *Toxocara* eggs, which are present in soil contaminated by dog/cat feces, or via the ingestion of larvae in undercooked meat [2]. Clinical manifestations of human toxocariasis are mostly asymptomatic; however, severe disease can occur when larvae migrate within the body to internal organs or the eyes, causing visceral and ocular larva migrans, neurotoxocariasis, and covert/common toxocariasis [1, 4].

Humans are incidental hosts for *Toxocara* spp. The larvae cannot develop into adult worms in the human small intestine; thus, no *Toxocara* eggs are present in human stool [1, 5]. The typical diagnosis of toxocariasis usually relies on serological tests. An enzyme-linked immunosorbent assay (ELISA) using *Toxocara* excretory-secretory (TES) antigens has been widely used and is recommended by the Centers for Disease Control and Prevention for the detection of *Toxocara*-specific IgG [6, 7]. The limitations of this test are its cross-reactivity with other parasites, especially the human roundworm *Ascaris lumbricoides* [8], and its lack of ability to differentiate between active and past infections. However, ELISA remains useful in seroepidermiological surveys, as it is a rapid and affordable method of determining the prevalence of asymptomatic toxocariasis.

Seroprevalence studies have been performed in several countries around the world. In some parts of the world, such as Nigeria (86.1%) [9], the Republic of the Marshall Islands (86.75%), [10] and Northeast Brazil (63.6%) [11], the seroprevalence of toxocariasis was remarkably high. In Asia, the seroprevalence of toxocariasis was substantially high: 46.0% in Taiwan [12], 45.9% in Turkey [13], 49% in the Philippines [14], 51.2% in Korea, and 23.5–45.9% in Iran [15, 16]. In Western countries, the prevalence was 5.0% in the USA [17], 8.0% in Italy [18], and 16.0% in Greece [19]. A systematic review and meta-analysis revealed that the global seroprevalence of toxocariasis was 19.0%, with the highest rate in the African region (37.7%) and the lowest rate in the Eastern Mediterranean region (8.2%) [20]. Many factors have been proposed to be involved, including contact with dogs [11, 15], children’s age [21], and male sex [11, 22, 23].

In Thailand, previous reports revealed that *Toxocara* eggs were found in the stool of dogs and cats [24, 25]. Furthermore, *Toxocara* eggs were identified in raw vegetables from markets in southern Thailand [26]; however, their impact on humans has not been investigated. In this study, we aimed to assess the seroprevalence of *Toxocara canis* infection and the associated risk factors among schoolchildren in rural areas of southern Thailand.

## Methods

### Study design and setting

**Fig. 1:**
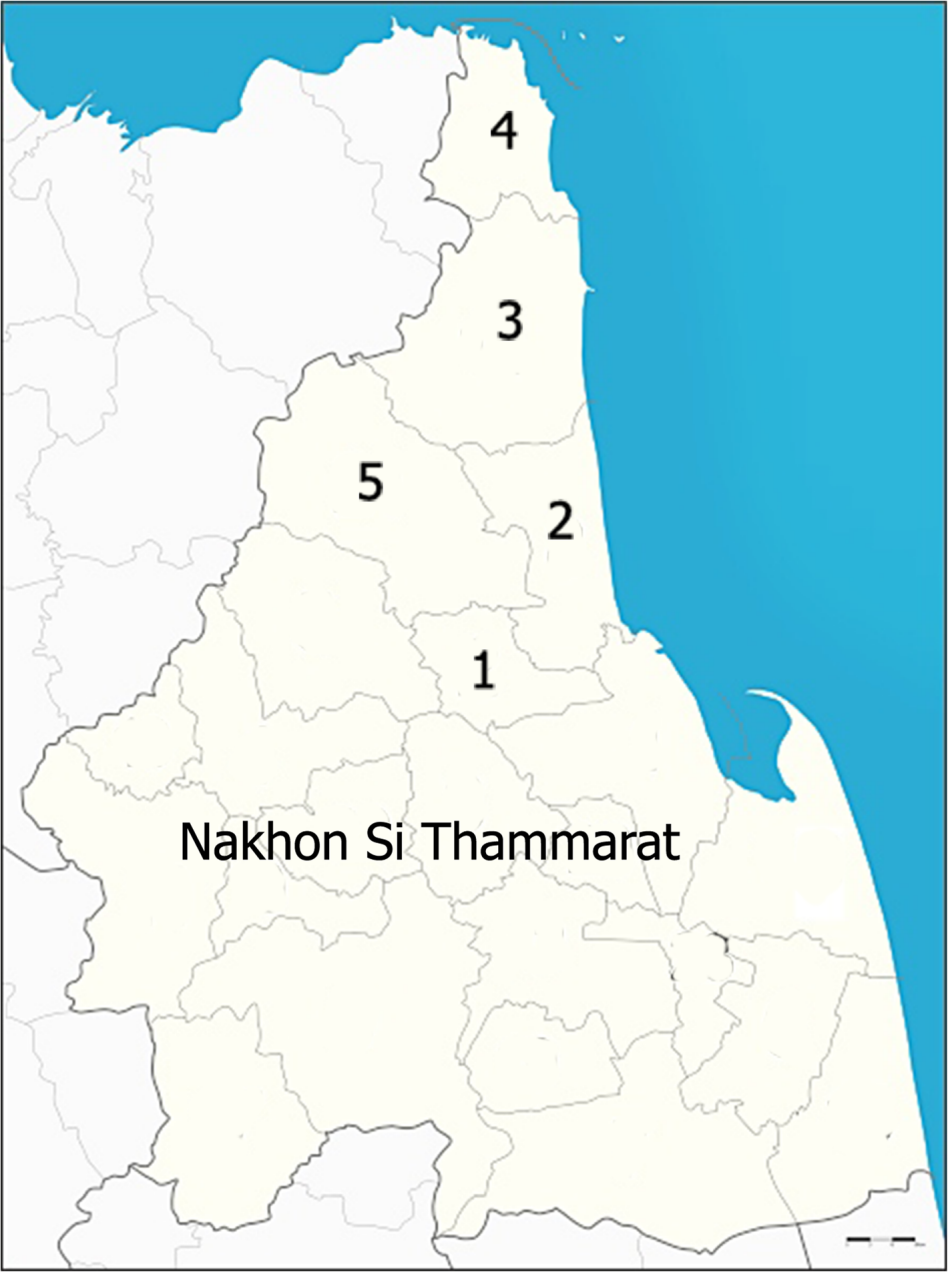
Map of Phrom Khiri (1), Tha Sala (2), Sichon (3), Khanom (4), and Nopphitam (5) Districts, Nakhon Si Thammarat Province, southern Thailand. (Map from Wikimedia Commons: https://commons.m.wikimedia.org/wiki/Atlas_of_Thailand#)

This cross-sectional study was carried out from June to July 2019 in five districts of Nakhon Si Thammarat Province, including Phrom Khiri, Tha Sala, Sichon, Khanom, and Nopphitam. Nakhon Si Thammarat Province is located in southern Thailand, approximately 780 km from the Thai capital of Bangkok (8°25′7″ N 99°57′49″ E) (Fig. 1). The average temperature is 27.1 °C, with a low temperature of 25.8 °C in January and a high temperature of 29.3 °C in May. The annual rainfall is 1454.3 mm (Climatological Center, Thai Meteorological Department). The Thailand Department of Provincial Administration estimates that the total populations of the subdistricts were 27,302 in Phrom Khiri, 113,323 in Tha Sala, 81,888 in Sichon, 20,369 in Khanom, and 33,543 in Nopphitam. The five districts are similar in terms of culture, economic status, and climate.

### Study population and sample size

The study population consisted of primary schoolchildren from 7 to 13 years of age. The sample size was determined using the single proportion population formula:

$$ {Z}^2p\left(1-p\right)/{d}^2 $$

where *p* = prevalence of intestinal parasites from a previous study, *d* = margin of error, and *Z* = standard score, which corresponds to 1.96. This formula was calculated based on a prevalence rate of 86.75% from a previous study [10], with a margin of error of 0.05 and a confidence level of 95%. The calculated sample size was 177. The exclusion criteria were immune system disorders (such as autoimmune disorders and acquired immune deficiency syndrome), steroid treatment for at least 3 months and acute illnesses on the day of blood collection. Three schools were randomly selected from each district for a total of 15 schools. Finally, the participants were selected from the school rosters using a systematic random sampling technique in which every 10th person was included in the study.

### Questionnaire survey

A structured questionnaire was developed and used to collect demographic data (i.e., age, sex, and parental education and occupation) and information on possible risk factors (habits of handwashing before a meal, after playing and after animal contact; owning pets (dogs or cats); eating fresh vegetables; and drinking untreated water). Agriculturists, farm laborers, housemaids, fishing boat staff, and street vendors were considered non-skilled workers. The questionnaire was administered by two trained interviewers who conducted direct interviews with the participating schoolchildren.

### Blood collection and preparation

Blood samples were collected from the antecubital vein by medical technologists and kept for 45 min at room temperature. After that, serum was separated by centrifugation at 2500 rpm for 10 min and stored at – 80 °C until the measurement of IgG class antibodies against *T. canis*.

### Detection of anti-*T. canis* IgG antibodies

Serum anti-*T. canis* IgG antibodies were detected with a commercial ELISA kit (ab108775, Abcam, UK), which has > 95% sensitivity and specificity according to the manufacturer’s instructions. In brief, all samples were diluted to 1:100 with IgG sample diluent, and all controls (*T. canis* IgG-positive, *T. canis* IgG-negative, and *T. canis* IgG cutoff (CO)) were prepared. The absorbance values were 0.150–1.300 for CO, < 0.200 and < cutoff for negative controls and > cutoff for positive controls. A precoated plate was incubated (100 μL/well) with the control or diluted samples for 1 h at 37 °C. After incubation with the serum samples, the plates were washed 3 times with ×1 washing solution and incubated (100 μL/well) with protein A HRP conjugate for 30 minutes at room temperature. The plates were washed 3 times and incubated (100 μL/well) with TMB substrate solution for exactly 15 min at room temperature in the dark. The reaction was stopped (100 μL/well) with stop solution for 15 min at room temperature. The assay included negative and positive serum samples in addition to a blank (no serum sample). Absorbance at 492 nm was measured using an automatic microplate reader. For interpretation of the results, samples with an absorbance value of more than 10% above the CO were considered positive. If the absorbance value was between 10% above and 10% below the CO, it was considered inconclusive (in the gray zone), and a fresh sample was run again. If the results of the second test were less than 10% above or below the CO control value, the sample was considered negative.

### Data analysis

Data were entered, cleaned, and analyzed using IBM SPSS Statistics for Windows, Version 23. Quantitative variables are described as the medians and interquartile ranges (IQRs), and qualitative variables are described as the frequencies (percentages). A chi-square test was used to compare the proportions of *T. canis* infection among subgroups stratified by sex, age group, level of education, district, parental occupation, and parental education. A univariate logistic regression model was constructed to investigate risk factors associated with *T. canis* infection. The variables with *P* values less than 0.2 in the univariate logistic regression model were included in a multiple logistic regression model that was adjusted for confounding factors. Differences were considered to be statistically significant when the *P* value obtained was less than 0.05.

## Results

### Sociodemographic characteristics

A total of 177 schoolchildren (91 boys and 86 girls) from 5 districts were included in this study. The median age of the study population was 9.82 years (IQR 9–11 years). The enrolled schoolchildren resided in 5 districts of Nakhon Si Thammarat, including Khanom (*n* = 35), Sichon (*n* = 34), Phrom Kiri (*n* = 34), Nopphitam (*n* = 36), and Thasala (*n* = 38). Their parents’ occupation and education level were mostly non-skilled workers and high school or less, respectively (Table 1).

**Table 1:** Sociodemographic characteristics of 177 schoolchildren in 5 districts of Nakhon Si Thammarat, Thailand

Characteristics		Seropositive		
		Number	%	*P* value
Sex	Male (*n* = 91)	59	64.8	0.065
	Female (*n* = 86)	44	51.2	
Age group (years)	6–8 (*n* = 32)	18	56.3	0.970
	9–10 (*n* = 82)	48	58.5	
	11–13 (*n* = 63)	37	58.7	
Level of education (grade)	1 (*n* = 15)	8	53.3	0.256
	2 (*n* = 17)	11	64.7	
	3 (*n* = 41)	28	68.3	
	4 (*n* = 32)	14	43.8	
	5 (*n* = 32)	16	50.0	
	6 (*n* = 40)	26	65.0	
Districts	Khanom (*n* = 35)	19	54.3	0.672
	Sichon (*n* = 34)	18	52.9	
	Phrom Kiri (*n* = 34)	20	58.8	
	Nopphitam (*n* = 36)	20	55.6	
	Thasala (*n* = 38)	26	68.4	
Father’s occupation	Non-skilled workers (*n* = 165)	98	59.4	0.229
	Skilled workers (*n* = 12)	5	41.7	
Mother’s occupation	Non-skilled workers (*n* = 163)	96	58.9	0.517
	Skilled workers (*n* = 14)	7	50.0	
Father’s education	High school or less (*n* = 160)	96	60.0	0.135
	Bachelor’s degree or more (*n* = 17)	7	41.2	
Mother’s education	High school or less (*n* = 159)	96	60.4	0.080
	Bachelor’s degree or more (*n* = 18)	7	38.9	
Total (*n* = 177)		103	58.2	

### Seroprevalence of *Toxocara canis* infection

The overall seroprevalence of *T. canis* infection among the participants was 58.2% (95% CI 50.9–65.5%). Boys had a higher seropositive rate (64.8%) than girls (51.2%). The rates of seropositivity (54.3% in Khanom, 52.9% in Sichon, 58.8% in Phrom Kiri, 55.6% in Nopphitam, and 68.4% in Thasala) were not significantly different among the 5 districts (*P* = 0.672). The highest seropositivity was observed among third-grade students (68.3%), followed by sixth-grade students (65.0%), while the lowest rate was observed among fourth-grade students (43.8%). Chi-square tests showed that there were no significant associations between *T. canis* infection and sex, age group, level of education, district, parents’ occupation and parents’ education (Table 1).

### Associated risk factors for *Toxocara canis* infections

The univariate analysis revealed that handwashing before a meal, handwashing after animal contact, and drinking untreated water were significantly associated with seropositivity for *T. canis*. However, the multivariate analysis indicated that handwashing before a meal was the only factor associated with *T. canis* seropositivity. Children who did not practice handwashing before a meal were more likely to be infected with *T. canis* than those who did (AOR = 2.20, 95% CI 1.11–4.34) (Table 2).

**Table 2:** Factors associated with *T. canis* infection among primary schoolchildren from rural areas of southern Thailand

Variables		Number (%)	No. positive (%)	No. negative (%)	COR	95% CI	*P* value	AOR	95% CI	*P* value
Age (years)	6–8	32 (18.1)	18 (56.3)	14 (43.8)	1					
	9–10	82 (46.3)	48 (58.5)	34 (41.5)	1.10	0.48-2.51	0.824			
	11–13	63 (35.6)	37 (58.7)	26 (41.3)	1.11	0.47-2.62	0.817			
Sex	Male	91 (51.4)	59 (64.8)	32 (35.2)	1.76	0.96-3.22	0.066	1.04	0.57–1.89	0.910
	Female	86 (48.6)	44 (51.2)	42 (48.8)	1			1		
Handwashing before a meal	Yes	224 (74.9)	19 (8.5)	205 (91.5)	1			1		
	No	75 (25.1)	13 (17.3)	62 (82.7)	3.67	1.93-6.95	< 0.001*	2.20	1.11–4.34	0.023*
Handwashing after playing	Yes	102 (57.6)	56 (54.9)	46 (45.1)	1					
	No	75 (42.4)	47 (62.7)	28 (37.3)	1.38	0.75-2.54	0.301			
Handwashing after animal contact	Yes	101 (57.1)	48 (47.5)	53 (52.5)	1			1		
	No	76 (42.9)	55 (72.4)	21 (27.6)	2.89	1.53-5.47	0.001*	1.686	0.83–3.44	0.150
Owning dogs	Yes	71 (40.1)	37 (52.1)	34 (47.9)	1.52	0.83-2.79	0.180	1.129	0.53–2.41	0.754
	No	106 (59.9)	66 (62.3)	40 (37.7)	1			1		
Owning cats	Yes	73 (41.2)	46 (63.0)	27 (37.0)	1.41	0.76-2.59	0.277			
	No	104 (58.8)	57 (54.8)	47 (45.2)	1					
Frequency of playing with dogs	Never	72 (40.7)	37 (51.4)	35 (48.6)	1			1		
	1–2 times/week	53 (29.9)	34 (64.2)	19 (35.9)	1.69	0.82-3.50	0.156	1.012	0.45–2.27	0.978
	3–4 times/week	22 (12.4)	16 (72.7)	6 (27.3)	2.52	0.89-7.18	0.083	1.258	0.37–4.26	0.712
	5–7 times/week	30 (16.9)	16 (53.3)	14 (46.6)	1.08	0.46-2.54	0.858	0.599	0.21–1.68	0.330
Eating fresh vegetables	Yes	36 (20.3)	19 (52.8)	17 (47.2)	1.32	0.63-2.75	0.461			
	No	141 (79.7)	84 (59.6)	57 (40.4)	1					
Drinking untreated water	Yes	113 (63.8)	72 (63.7)	41 (36.3)	1.87	1.00-3.48	0.049*	1.429	0.69–2.96	0.338
	No	64 (36.2)	31 (48.4)	33 (51.6)	1			1		

*COR* crude odds ratio, *AOR* adjusted odds ratio, *CI* 95% confidence interval

*Significant association

When stratified by age, the rates of seropositivity for *T. canis* were approximately equal among age groups (56.3%, 58.5%, and 58.7% for children 6–8 years of age, 9–10 years of age and 11–13 years of age, respectively). Boys were more likely to be infected with *T. canis* than girls. Children who did not practice handwashing after playing, children who owned dogs and cats and children who ate fresh vegetables were more likely to be infected with *T. canis* than their counterparts*.* However, there was no statistical significance among these factors in either the univariate or multivariate analyses (Table 2).

## Discussion

Toxocariasis is a prevalent parasitic zoonosis worldwide, especially in the tropics and subtropics. This study was the first serological investigation of *T. canis* infection among schoolchildren in southern Thailand, with a prevalence rate of 58.2%, which was higher than the global seroprevalence (19.0%) and the pooled seroprevalence in Southeast Asia (34.1%) [20]. The rate of *T. canis* infection in this study was slightly higher than the rates in other countries in Asia, including the Philippines (49.0%) [14], Taiwan (57.5%) [27], Turkey (45.9%) [13], Korea (51.2%) [28], and Isfahan, Iran (45.9%) [16], whereas the rate was lower than those in some other parts of the world, including Nigeria (86.1%) [9] and the Republic of the Marshall Islands (86.8%) [10]. In general, multiple factors, including environmental, geographic, cultural, and socioeconomic factors, contribute to the prevalence of *T. canis* infection. Our study areas included five districts of Nakhon Si Thammarat Province, all of which were considered rural. Most of the countryside in these districts contains rubber plantations and farmlands, and dogs are commonly owned as pets; moreover, there is a large number of stray dogs in these rural districts. Nakhon Si Thammarat has a tropical rainforest climate; the temperature ranges from 24 to 34 °C, which is suitable for the development of *Toxocara* eggs to the infective larval stage [29]. We hypothesized that climate, soil and the number of dogs in the areas influenced the rate of seropositivity in this study.

Among the five districts, Thasala showed the highest rate of *Toxocara* seropositivity (68.4%); however, there was no statistically significant difference among the five districts. This might be explained by the similarities in climate, geography, and culture among the districts. Children whose parents were non-skilled workers and had low education levels had a relatively higher rate of *Toxocara* seropositivity. This trend was also observed in a previous study in the Republic of the Marshall Islands [10]. However, parents’ education levels and occupations did not significantly affect the seroprevalence rate in this study. This might be due to a small number of children whose parents were skilled workers and had obtained college degrees.

Among the age groups, there was no significant difference in the rate of *Toxocara* seropositivity in the current study. The rates of *Toxocara* seropositivity were approximately similar across all age groups: 56.3% in children 6–8 years of age, 58.5% in children 9–10 years of age, and 58.7% in children 11–13 years of age. Similar findings were observed in the study from the Republic of the Marshall Islands [10]. However, this result was in contrast to the results of several studies among schoolchildren that showed that older age was a significant risk factor for *T. canis* infection [9, 15, 22, 30, 31]. The detection of *Toxocara* via serology increases over time because of the persistence of serum IgG. The possible explanation for the similar seropositivity rates among the different age groups might be that the children might have been in contact with *Toxocara* eggs since a young age, and the IgG persists over a long period of time. Our study revealed that boys tended to be infected with *Toxocara* spp. more often than girls (64.8% in boys and 51.2% in girls). This trend was also observed in previous studies [11, 22, 23]; it has been hypothesized that boys engage in more outdoor activities than girls; thus, they are more likely to be in contact with soil and dogs. However, sex was not significantly associated with *Toxocara* seropositivity in either the univariate or multivariate analysis in the current study.

Regarding modifiable risk behaviors, handwashing before a meal appeared to be the only significant risk factor associated with *Toxocara* infection in both the univariate and multivariate analyses. Schoolchildren who did not practice handwashing before a meal were 2.2 times more likely to be infected with *T. canis* than those who did. This effect was also observed in a previous study among Brazilian schoolchildren [32]. Furthermore, the univariate analysis in this study revealed that handwashing after animal contact and drinking untreated water were significantly associated with *T. canis* infection; however, these associations were not statistically significant in the multivariate analysis. Humans acquire *T. canis* by ingesting infective eggs, which can be found in public places worldwide, including Thailand [33]. This study emphasizes the importance of hand hygiene to prevent parasite eggs from entering the body. Despite the report of *Toxocara* egg contamination in raw vegetables in Nakhon Si Thammarat Province in a previous study [26], eating fresh vegetables was not significantly associated with *T. canis* infection in the current study. This may be explained by the small number of schoolchildren who ate fresh vegetables (36, 20.3%); thus, contamination from vegetables was not a likely route of transmission among these children. Schoolchildren who engaged in risky behaviors, such as owning dogs and owning cats, also tended to have a higher rate of *T. canis* infection than those who did not; however, no significant associations were observed among these factors and *T. canis* infection.

This study had the following limitations. First, the method for the detection of anti-*T. canis* IgG antibodies was an ELISA, which is not the gold standard test, *Toxocara* spp. larval excretory-secretory Western blot, and could yield false positive results due to cross-reactivity with other helminths, especially *A. lumbricoides* [8, 34]. However, our study sites were not endemic areas for *A. lumbricoides* [35, 36]; hence, the chance of false positivity was likely low. Second, the study design was cross-sectional, and seroprevalence and risk factors were evaluated simultaneously, not over a period of time; thus, true causes and effects might not be strongly demonstrated.

## Conclusions

This is the first serological investigation of *T. canis* infection among schoolchildren in Thailand. The high rate of *Toxocara* seropositivity reflected high levels of exposure to *T. canis* among schoolchildren in rural areas of southern Thailand. A lack of handwashing before meals appeared to be a significant risk factor for *T. canis* infection. Appropriate public health education on personal hygiene, especially strengthening hand hygiene practices among schoolchildren, should be implemented to prevent the transmission of *T. canis*.

## Data Availability

All data generated or analyzed during this study are available from the corresponding author on reasonable request.

## Abbreviations

AOR: Adjusted odds ratio
CI: Confidence interval
CO: Cutoff
COR: Crude odds ratio
IQR: Interquartile range
